# Spatiotemporal Variations and Determinants of Under-Five Stunting in Ethiopia

**DOI:** 10.1177/03795721231158503

**Published:** 2023-02-23

**Authors:** Fikrewold H. Bitew, Corey S. Sparks, Samuel H. Nyarko, Lauren Apgar

**Affiliations:** 1 Department of Demography, College for Health, Community & Policy, The University of Texas at San Antonio, San Antonio, TX, USA; 2 Institutional Research, The University of Texas at San Antonio, One UTSA Circle, San Antonio, TX, USA; 3 Department of Epidemiology, Human Genetics & Environmental Sciences, School of Public Health, The University of Texas Health Science Center at Houston (UTHealth), Houston, TX, USA

**Keywords:** spatial, temporal, under-five stunting, Ethiopia

## Abstract

**Background:**

Stunting has been a major concern in sub-Saharan Africa. However, little evidence exists on the spatiotemporal variations in under-five stunting within a national context.

**Objective:**

This paper examines the spatiotemporal variations in under-five stunting and determinants using data from the Ethiopia Demographic and Health Surveys (2000-2016).

**Methods:**

Spatial autocorrelation and multilevel logistic regression models were used to conduct the analyses.

**Results:**

The stunting prevalence has decreased from 51% to 37%, while the prevalence of severe stunting has decreased by more than half (from 28% to 12%). Wide regional variations in stunting have been consistently observed over the years, which exhibited a higher level of stunting in Tigray (48%), Afar (42%), and Amhara (42%). The results show considerable local and regional variations in under-five stunting levels with diverse patterns of improvements in regional stunting levels over time. Stunting levels were associated with child-level factors such as the sex of a child, birth size, age of a child, birth order, preceding birth interval, and place of birth. Maternal educational attainment, nutritional status, household wealth, toilet facility type, and place of residence were linked to under-five stunting. The regional-level infant mortality rate was associated with under-five stunting.

**Conclusions:**

Specially tailored policies and interventions should be devised to address persistent spatial inequalities in stunting by focusing on higher risk populations.

## Introduction

Childhood stunting (low height-for-age) adversely affects the survival, growth, and development of children, impacting 149 million (21.9%) under-five children worldwide.^1^ Child stunting levels have shown steady improvement globally, except in Africa where improvement has slowed in recent years.^1,2^ Consequently, the World Health Organization’s (WHO) global target of a 40% reduction in the number of stunted children by 2025^3^ is unlikely to be met. In Ethiopia, stunting cases among under-five children have decreased from 58% in 2000 to 38% in 2016, a reduction of about a third,^4^ which leaves more work to be done.

Several long-term adverse effects including poor health, limited cognitive, physical, economic, and reproductive performance among others, have been widely linked to childhood stunting.^5^ To achieve the 2025 global target, WHO suggested that situational analysis be conducted to uncover geographic variation in childhood stunting and its underlying context-specific causes.^4^ Multiple studies have shown spatial and temporal differences in childhood undernutrition and its determinants in sub-Saharan Africa.^6,7^ Substantial spatiotemporal variations in chronic malnutrition among under-five children have been found in Ghana.^6^ Additionally, regions within the country have been identified as high-risk areas for chronic malnutrition.^6^ In Cote d’Ivoire, temporal changes in stunting among under-three children between 1994 and 2011 have been documented, along with spatial differences at the national and subnational levels.^7^ These previous studies have indicated that age, sex, household wealth, maternal education, and maternal body mass index are significantly associated with under-five stunting,^6-8^ but regional-level factors were not examined.

Few previous studies have, however, considered the spatial aspect of stunting among children in Ethiopia.^8-10^ These studies did not consider the trends of stunting prevalence at the local and regional levels over time, as they were based on only one survey year^9^ while another focused on a specific part of the country and, therefore, had no national representation.^8^ Besides, none of these studies examined individual-level factors in combination with aggregate-level regional variables across regions and over time.

This study fills these literature gaps by drawing on nationally representative data to conduct a spatiotemporal analysis of under-five stunting in Ethiopia. The study is guided by 2 main research questions: (1) Are there spatial variations in child stunting in Ethiopia and do they change over time? (2) Are spatial and temporal variations in child stunting explained in part or in full by individual and regional contextual factors? The spatiotemporal analysis is relevant because it may have an additional advantage over separately analyzing spatial or temporal trends. The analysis identifies local areas of high risk, illuminates any unusual patterns and persistence of undernutrition disparities or clustering over time, and ultimately helps to direct tailored programs and interventions to address stunting disparities. The statistical analysis also provides a better understanding of the individual-level and regional contextual factors associated with spatiotemporal disparities in under-five stunting in Ethiopia.

## Data and Methods

### Data Source

This study draws on pooled cross-sectional data from the Ethiopian Demographic and Health Surveys (EDHS) (2000-2016) which are part of the global demographic and health survey (DHS) series that are collected at regular periods.^4,11-13^ These data sets are comparative and provide a reliable source of maternal and child health data including height-for-age (stunting) data at the individual level. Ethiopian Demographic and Health Surveys are nationally representative data that used a multistage sampling procedure where respondents were selected from households in randomly selected clusters in both rural and urban settings in the country. The National Ethics Review Committee of Ethiopia reviewed and approved the EDHS protocol. The study sample comprised 8890, 4363, 10 222, and 9341 children under 5 years of age for the years 2000, 2005, 2011, and 2016, respectively, for a total sample of 32 816.

### Outcome Variable

The outcome variable is stunting as determined by height-for-age among children under the age of 5. Height-for-age is a measure of linear growth retardation and cumulative growth deficits. Based on the WHO growth standards, children whose height-for-age Z-score is negative 2 standard deviations or more (−2 SD) from the median of the reference population are considered stunted.^14^ We use the Z-scores of height-for-age in the dataset to create a dichotomous measure (1 = stunted, 0 = not stunted) based on these standards.

### Predictors and Measurements

The study examined numerous individual-level sociodemographic predictors that were selected based on their significant association with under-five stunting in the extant empirical literature.^15-18^ Thus, variable selection and measurements are mainly supported by the existing literature. The sex of a child (male/female) was included in the study, while the age of a child was measured in months as < 12, 12-23, 24-35, 36-47, and 48-59. Child size at birth was measured in 3 categories such as small, average, and large. The preceding birth interval was measured as < 2 years, 2-4 years, and > 4 years, while birth order number was measured as 1 to 2, 2 to 4, and 5+. Child’s place of delivery was measured as a home or health facility.

Several maternal factors were as well included in the study. Maternal age at birth was measured as < 20 years and 20 years or more, and maternal educational attainment was measured as no education, primary education, and secondary education or higher. Maternal employment status was recoded as employed or not employed, while maternal nutritional status was recoded as underweight or not underweight, and maternal breastfeeding status was measured as ever breastfed or never breastfed. Household wealth status was based on a wealth index collapsed into poor, middle, and rich, while the source of drinking water and type of toilet were both measured as improved or unimproved. Rural versus urban places of residence were also included in the study.

Also, some regional aggregate level factors were included in the study to assess regional effects on stunting at the child level. We considered the median duration of exclusive breastfeeding by women in the region, the percentage of children with diarrhea who received an oral rehydration solution or recommended homemade fluids treatment, the percentage of married women with an unmet need for family planning, the region’s infant mortality rate, and the percentage of households in the region with electricity. These aggregate-level variables were generated from the DHS for aggregate-level analysis and are all included as continuous measures.

### Analytic Approach

The R statistical package (version 3.6.3) was used for all data processing and analysis.^19^ Two main analyses were performed. The first level comprised the analysis of the background characteristics of respondents and the spatialtemporal variations in stunting using geospatial methods. In the spatial data analysis, DHS global positioning system (GPS) data points were used to generate spatial weights at the primary sampling unit (cluster) level using the 3-nearest neighbors rule for each of the survey years. Voronoi polygons were used to create buffers around neighboring GPS points. We used the 3-nearest neighbor rule as it provided the best clustering patterns during the exploratory analysis. The spatial weights were attached to the study data to calculate Moran’s I statistic to assess potential spatial autocorrelation of stunting at the cluster level. The Moran’s I analysis generated positive results indicating spatial clustering of stunting. Local Moran’s I analysis was further conducted to generate Z-scores whereby positive values represent “high” clustering while negative values represent “low” clustering of stunting. The results were then attached to shapefiles and plotted as cluster maps showing hot spots and cold spots of stunting for all the years. A logistic regression model was used to produce regional estimates of stunting which were used to create regional maps and geometric lines to visualize the spatial and temporal trends of stunting.

For the second analysis, multilevel logistic regression was used to examine the association between individual and region-level predictors on under-five stunting. We specified the models at two levels, where children at the individual level were nested within regions (higher level hierarchy) over time, taking into consideration both individual and aggregate-level predictors. This can be mathematically specified as:


$$\text{logit}\left(Pr\left(\text{stunting}\right)\right)={\text{β}}_{0j}+\sum {\text{β}}_{k}x_{k}+{\text{γ}}_{j}z_{jt}+vj\text{ * }Year$$

$$\begin{array}{c} {\text{β}}_{0j}=\;{\text{β}}_{0}+u_{j},\text{ with }u_{j}∼\text{Normal }\left(0,\;\sigma_{u}\right)\\ {\text{ν}}_{j}=v_{0}+v_{j},\text{ with }v_{j}∼\text{ Normal }\left(0,\;\sigma_{v}\right) \end{array}$$

Where *j* represents the region of residence of the children, while $\beta_{0j}$ represents the region’s random intercept term. The $\beta_{k}x_{k}$ term is the regression effect of the individual-level predictors while the $\gamma z_{jt}$ term represents the regional aggregate-level predictors, which also change over time *t*. The *Year* term is the overall time trend within the country while the $\nu_{j}$ term is the random slope for the time trend within each region.

Three models were estimated. As a baseline model, Model 1 was specified to examine the temporal effect on stunting among the regions by using only time random slopes and the regional level random intercepts without the individual and region-level predictors. Model 2 adjusted for individual-level sociodemographic factors, while Model 3 further controlled for regional aggregate-level predictors. Due to the multistage sampling procedure used for selecting respondents, the analysis was weighted using complex survey weights created by nesting the primary sampling unit (clusters) and the strata (residence) in the regions to address potential over-sampling and under-sampling in the study sample and reduce biased estimates. The model parameters were used to calculate odds ratios (OR) and 95% confidence intervals (CI).

## Results

### Descriptive Results

Table 1 presents the prevalence of stunting across the survey years and Ethiopian regions, as well as across child, maternal, and household characteristics. The national aggregate level of stunting reduced significantly from 51.4% in 2000 to 33.0% in 2016. Among the 32 816 under-five children in the sample, stunting was more common for male children (42.7%) than females (40.6%). Stunting was more prevalent among children having a small size at birth (44.9%) than children born at an average (39.8%) or large size (38.9%). Stunting was considerably higher among older children aged 36 to 47 (50.9%) and 48 to 59 (49.6%) months than in children less than 1 year (14.4%).

**Table 1.** Descriptive Statistics of Childhood Stunting Outcome by Study Characteristics, EDHS 2000-2016.^a^

Background characteristics	Stunted before age 5	Chi-square test of equality
	Percent	
Year		***P* < .0001**
2000	51.4	
2005	46.4	
2011	38.6	
2016	33.0	
Sex of a child		***P* < .0061**
Female	40.6	
Male	42.7	
Child size		***P* < .0001**
Small	44.9	
Average	39.8	
Large	38.9	
Age of child in months		***P* < .0001**
0-11	14.4	
12-23	47.8	
24-35	45.9	
36-47	50.9	
48-59	49.6	
Birth order		***P* < .0001**
1st-2nd	39.0	
3rd or later	43.1	
Preceding birth interval		***P* < .0001**
<2 years	48.6	
2-4 years	42.8	
>4 years	35.7	
Place of delivery		***P* < .0001**
Home	43.7	
Health facility	30.9	
Age of mother at first birth		*P* = .0621
<20 years	42.2	
20+ years	40.6	
Mother’s educational level		***P* < .0001**
No education	44.8	
Primary	36	
Secondary/above	21.5	
Mother’s employment status		*P* = .7151
Employed	41.5	
Not employed	41.8	
Mother’s nutritional status		***P* < .0001**
Underweight	45.0	
Not underweight	40.8	
Breastfeeding status		***P* < .0001**
Ever breastfed	41.7	
Never breastfed	36.9	
Wealth index		***P* < .0001**
Poor	44.4	
Middle	40	
Rich	36.2	
Source of drinking water		***P* < .0001**
Improved	37.6	
Unimproved	44.8	
Type of toilet facility		***P* < .0001**
Improved	32.2	
Unimproved	42.7	
Type of place of residence		***P* < .0001**
Rural	43.1	
Urban	29.5	
Region		***P* < .0001**
Tigray	44.0	
Afar	42.3	
Amahara	49.4	
Oromiya	38.0	
Somali	30.7	
Benshangul-Gumuz	40.5	
SNNPR	44.0	
Gambella	26.1	
Harari	30.7	
Addis Ababa	17.1	
Dire Dawa	32.7	

Abbreviations: EDHS, Ethiopian Demographic and Health Surveys; SNNPR, Southern Nations, Nationalities, and Peoples’ Region.

Boldface values Significance <0.05.

^a^ N = 32 816.

Children of higher birth order (3rd or higher; 43.1%), children with a preceding birth interval of less than 2 years (48.6%), and children born at home (43.7%) had significantly higher levels of stunting compared to their respective counterparts. There were also significantly higher stunting levels among children whose mothers had no education (44.8%), were underweight (45.0%), ever breastfed (41.7%), and lived in a poor household (44.4%). Children in households with unimproved drinking water (44.8%) and toilet facility (42.7%) also had considerably higher stunting levels than their counterparts with improved conditions (37.6% and 32.2%, respectively). Additionally, children who resided in rural areas (43.1%) were found to have higher stunting levels than their urban counterparts. High levels of regional variation in stunting are observed, with the highest levels of stunting found among children residing in the Amhara (49.4%), Tigray (44.0%), and the Southern Nations, Nationalities, and Peoples’ Region (44.0%), and the lowest prevalence (17.1%) found in Addis Ababa.

### Spatial and Temporal Disparities in Under-Five Stunting

**Figure 1.**
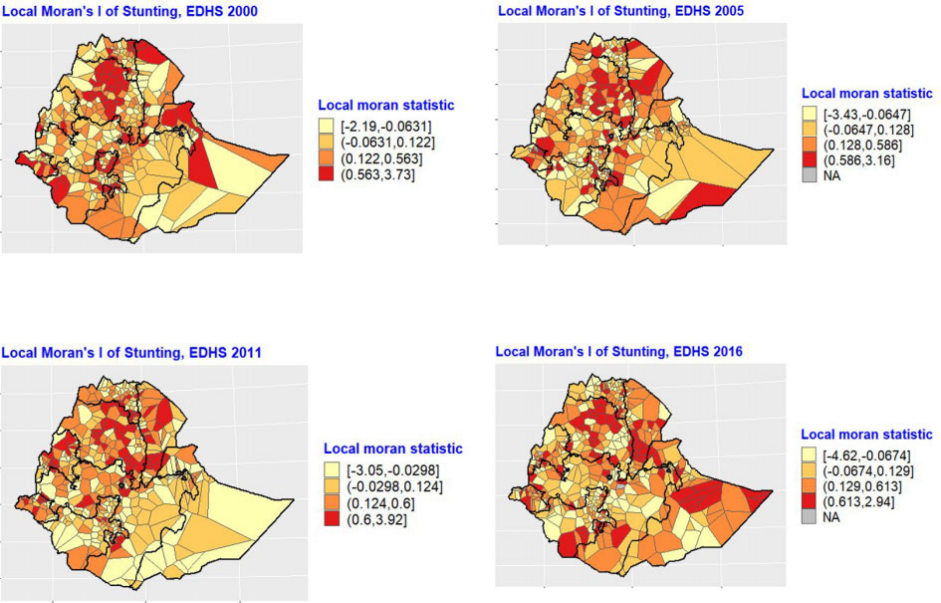
Local Moran’s I statistics (z-scores) of under-five stunting in Ethiopia, 2000-2016. Source: Created by the authors based on EDHS data.

The analysis generated Moran’s I statistics of 0.46 (*P* < .0001), 0.47 (*P* < .0001), 0.48 (*P* < .0001), and 0.49 (*P* < .0001) for the years 2000, 2005, 2010, and 2016, respectively, suggesting significant spatial clustering of stunting. Figure 1 is a cluster map of under-five stunting from a local Moran’s I analysis at the cluster level in z-scores. The positive values (red) indicate a clustering of high stunting (hot spots) while the negatives (yellow) indicate a clustering of low stunting (cold spots). The Amhara region in the northern part of the country consistently had several clusters of high stunting from 2000 to 2016. A few local clusters of high-level stunting were also found in a few other regions while pockets of low-stunting clusters can also be found in the southeastern part of the country.

**Figure 2.**
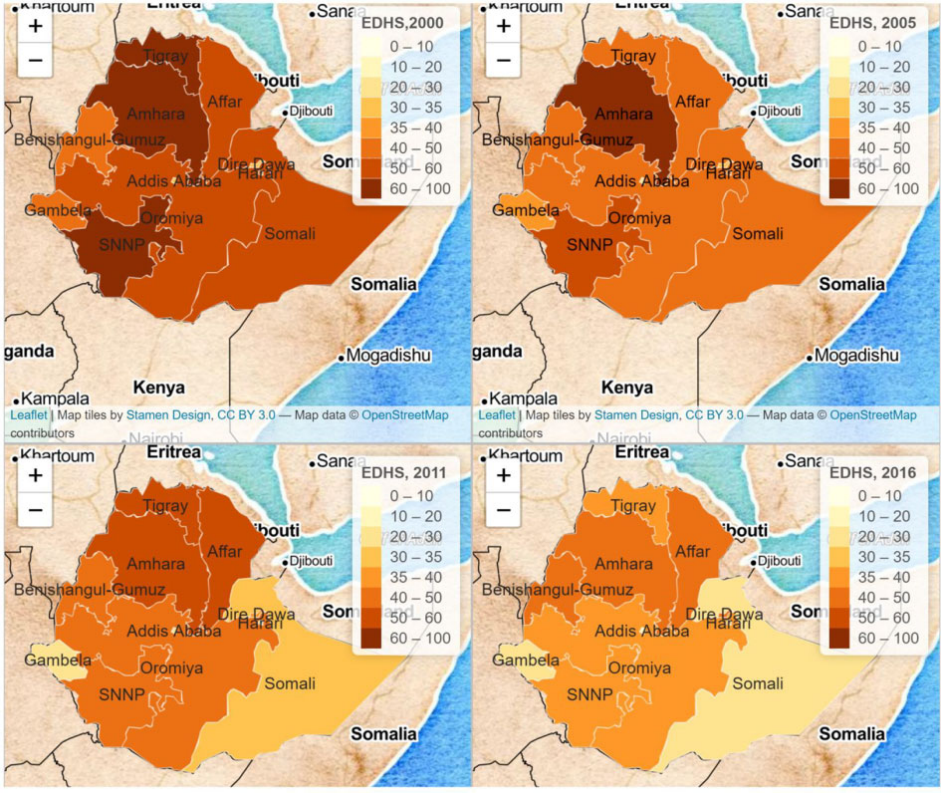
Spatiotemporal variations of under-five stunting levels in Ethiopia, Ethiopian Demographic and Health Surveys (EDHS) 2000-2016. Source: Created by the authors based on EDHS data.

**Figure 3.**
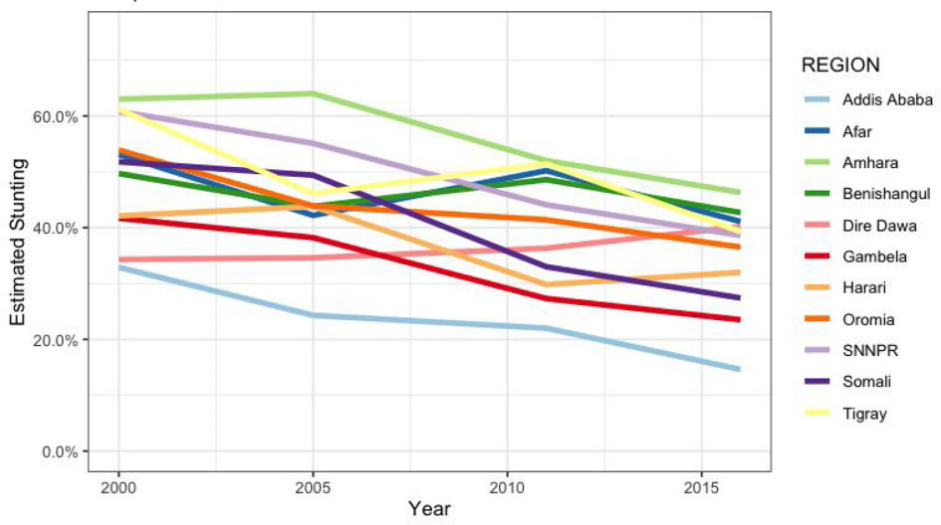
Temporal trends of regional under-five stunting levels in Ethiopia, Ethiopian Demographic and Health Surveys (EDHS) 2000-2016.

Figures 2 and 3 show the regional and temporal variations of children’s stunting prevalence in Ethiopia from the year 2000 to 2016. In 2000, the prevalence of stunting was highest in Amhara, Tigray, and SNNPR (63%, 61%, and 61%, respectively). In 2005, high levels of stunting persisted in Amhara and SNNPR (64% and 55%, respectively) and declined in Tigray. In 2011, the highest prevalence of stunting remained in the Amhara region (46%). Other northern regions proximate to Amhara, Affar, and Tigray had similarly high prevalence (41% and 40%, respectively). In 2016, a similar pattern can be observed, with Amhara having the highest prevalence. Overall, stunting levels remained the highest in Amhara, even though diverse patterns of reductions were observed in most of the regions over the period (see Figure 3). Addis Ababa consistently maintained the lowest prevalence of stunting, and all regions except Dire Dawa showed a notable decrease in stunting levels between 2000 and 2016.

### Multivariate Analysis Results

Results from multilevel logistic regression analyses of under-five stunting in Ethiopia are shown in Table 2. As a baseline model, Model 1 estimates temporal effects on stunting using time as the only predictor variable but including regional-level random intercepts and time random slopes. This model shows that stunting has reduced over time, with the odds of under-five stunting decreasing by 25% (OR: 0.75; 95% CI: 0.73-0.76) with each survey year. After adjusting for individual-level characteristics and regional aggregate factors, Model 3 shows that the odds of under-five stunting decreased by at least 14% (OR: 0.86; 95% CI: 0.75-0.99) over the 15-year study period. The variance terms for the regions and time (survey year) within the regions indicate significant variations among the regions and in the time trends across regions similar to what is shown in Figure 3.

**Table 2.** Multilevel Logistic Regression Models of Under-Five Stunting Among Children in Ethiopia, EDHS 2000-2016.

Predictors	Model 1	Model 2	Model 3
	OR (95% CI)	OR (95% CI)	OR (95% CI)
Year	0.75 (0.73-0.76)^a^	0.75 (0.73-0.77)^a^	0.86 (0.75-0.99)^a^
**Child/Mother/Household characteristics**			
Sex of a child (Ref: Female)			
Male		1.14 (1.09-1.20)^a^	1.10 (1.01-1.15)^a^
Size of a child at birth (Ref: Small)			
Average		0.74 (0.70-0.78)^a^	0.74 (0.70-0.78)^a^
Large		0.68 (0.64-0.73)^a^	0.68 (0.64-0.73)^a^
Age of the child in months (Ref: <12)			
12-23		5.66 (5.20-6.17)^a^	6.12 (5.42-6.91)^a^
24-35		5.24 (4.81-5.71)^a^	6.12 (5.42-6.92)^a^
36-47		6.39 (5.87-6.96)^a^	6.83 (6.06-7.71)^a^
48-59		6.10 (5.60-6.65)^a^	6.50 (5.77-7.34)^a^
Birth order number (Ref: 1-2)			
3-4		1.16 (1.09-1.24)^a^	1.08 (0.98-1.18)
5+		1.19 (1.11-1.26)^a^	1.11 (1.02-1.21)^a^
Preceding birth interval (<2 years)			
2-4 years		0.86 (0.81-0.91)^a^	0.86 (0.81-0.91)^a^
>4		0.69 (0.64-0.75)^a^	0.69 (0.64-0.75)^a^
Place of delivery (Ref: Home)			
Health facility		0.90 (0.83-0.97)^a^	0.91 (0.84-1.02)
Age of the mother at first birth (<20 years)			
20+ years		1.03 (0.98-1.08)	1.03 (0.97-1.11)
Mother’s level of education (Ref: No education)			
Primary		0.89 (0.84-0.95)^a^	0.90 (0.83-0.99)^a^
Secondary/Higher		0.51 (0.44-0.59)^a^	0.56 (0.46-0.69)^a^
Mother’s nutritional status			
Underweight		1.13 (1.07-1.20)^a^	1.13 (1.07-1.24)^a^
Mother’s breastfeeding status (Ref: Ever breastfed)			
Never breastfed		0.85 (0.72-1.01)	0.86 (0.82-1.02)
Mother’s employment status (Ref: Not employed)			
Employed		1.02 (0.98-1.08)	0.97 (0.91,1.05)
Household wealth status (Ref: poor)			
Middle		0.86 (0.81-0.92)^a^	0.86 (0.81-0.92)^a^
Rich		0.82 (0.78-0.88)^a^	0.82 (0.77-0.87)^a^
Source of drinking water (Ref: Improved)			
Unimproved		0.95 (0.90-1.00)	0.95 (0.91-1.01)
Type of toilet facility (Ref: Improved)			
Unimproved		1.15 (1.05-1.27)^a^	1.15 (1.04-1.26)^a^
Place of residence (Ref: Rural)			
Urban		0.88 (0.80-0.98)^a^	0.82 (0.72-0.94)^a^
**Regional characteristics**			
Median duration of exclusive breastfeeding			1.04 (0.97-1.06)
Treatment of diarrhea: either ORS or RHF			0.97 (0.91-1.08)
Median age at first marriage [women]			1.09 (0.94-1.27)
Percent unmet need for family planning			0.97 (0.91-1.04)
Infant mortality rate			1.15 (1.01-1.26)^a^
Households with electricity			0.97 (0.84-1.14)
**Variance components**			
ICC	0.14 (0.07-0.41)	0.05 (0.03-0.19)	0.05 (0.02-0.20)
WAIC	43 858.6	40 370.32	40 374.74
Log-likelihood	−21 958.96	−20 330.66	−20 360.1

Abbreviations: CI, confidence intervals; EDHS, Ethiopian Demographic and Health Surveys; ICC, Interclass Correlation Coefficient; WAIC, Watanabe–Akaike Information Criterion; OR, odds ratios; ORS, oral rehydration solution; Ref, reference category; RHS, recommended homemade fluids.

^a^ Significance < 0.05.

Also, individual-level sociodemographic characteristics were significantly associated with under-five stunting—except for the age of the mother at first birth, mother’s employment status, source of drinking water, and mother’s breastfeeding status—after adjusting for regional aggregate factors in Model 3. Male children were at least 10% (OR: 1.10; 95% CI: 1.01-1.15) more likely to be stunted compared to female children. Average-sized (OR: 0.74; 95% CI: 0.70-0.78) and larger-sized children at birth (OR: 0.68; 95% CI: 0.64-0.73) had significantly lower odds of being stunted compared to the smaller size children at birth. The odds of stunting increased significantly with the age of the child, with the odds of stunting being 6.83 times among older children ages 36-47 months compared to children aged < 12 months (95% CI: 6.06-7.71). The results also showed that the odds of stunting increased significantly for higher birth order children, such as order 3-4 (OR: 1.16; 95% CI: 1.09-1.24) and order 5+ (OR: 1.19; 95% CI: 1.11-1.26) compared to first or second-born children, while the odds of stunting significantly decreased with longer preceding birth intervals (2-4 years, OR: 0.86 95% CI: 0.81-0.89; and > 4 years, OR: 0.69 95% CI: 0.64-0.75) compared to the shortest birth interval (< 2 years). Relative to home-delivered children, children delivered at a health facility had 10% lower odds of stunting (95% CI: 0.83-0.97).

Compared to children whose mothers had no education, children whose mothers had higher education levels with primary education or a secondary level of education had a least 10% (OR: 0.90; 95% CI: 0.83-0.99) or 44% (OR: 0.56; 95% CI: 0.46-0.69) lower odds of being stunted, respectively. Children of underweight mothers had 13% higher odds of being stunted (OR: 1.13; 95% CI: 1.07-1.24) compared to children of mothers who were not underweight. Children from the middle-income (OR: 0.86; 95% CI: 0.81-0.92) and rich households (OR: 0.82; 95% CI: 0.77-0.87) had lower odds of being stunted compared to children from poor households. Similarly, children from households with unimproved toilet facilities had higher odds (OR: 1.15; 95% CI: 1.04-1.26) of being stunted compared to households with improved toilet facilities. Children in urban areas had at least 18% lower odds (OR: 0.82; 95% CI: 0.72-0.94) of stunting compared to children who reside in rural areas. At the regional level, only infant mortality rate was associated with under-five stunting, with the odds being significantly higher in regions that had above-average infant mortality rates in the country over the study period (OR: 1.15, 95% CI: 1.01-1.26).

## Discussion

The current study presents findings of a spatial and temporal analysis of under-five stunting using a nationally representative sample of children under 5 years in Ethiopia. We found several clusters of both high and low stunting values at the local cluster level over time, suggesting considerable disparities in stunting with most of the hot spots existing in the northern part of the country. This may be explained by the recurrent drought and poor sociocultural feeding habits in these regions. Evidence of spatial variations in under-five stunting has also been established in various countries including Uganda,^20^ Papua New Guinea,^21^ India,^22^ and Ghana.^23^ Generally, there is notable evidence of a steady decline in the regional levels of under-five stunting for the 15-year study period although the levels remain high. Diverse patterns of improvement in the stunting prevalence occurred in the various regions over time. Despite the observed temporal improvements, there was evidence of a north-south divide in stunting levels which mainly disadvantaged the northern states (Amhara, Benishangul, Afar, and Tigray) and to the advantage of the southern and the central part of the country, particularly the Addis Ababa Region which is also the capital city of the country.

The prevalence of stunting in Ethiopia was estimated to be 37% in 2019; in the same year, the percentage of stunting was higher in Amhara (41%), Benshangul-Gumuz (41%), Tigray (48%), and Afar (42%) compared to the national estimate.^24^ According to the joint child malnutrition estimates, when the prevalence of stunting becomes 30% or more, it is considered very high or critical.^25^ Therefore, our observed prevalence of stunting in these regions (Amhara, Benshangul-Gumuz, Tigray, and Afar) has been in a serious range over the study period (2000-2016). A study indicated that conflict-driven displacements in Afar and Tigray regions have been aggravating hunger and malnutrition rates up over the years.^26^ Also, intensified conflict on the Tigray-Afar border in recent days is expected to force more communities from their homes and deeper into hunger.^26^ The same study also indicated that there is a higher shortage of safe and adequate water supply and limited access to health services in these regions in regions of Tigray and Afar regions,^26^ which ultimately contributes to the higher level of stunting among under-five children in these regions. A study has linked stunting to food diversity and the number of meals a child ate per day and being underweight in the Amhara region.^27^ Consequently, child nutrition intervention strategies should seriously consider food security, and dietary diversity, and be specifically targeted to residential locations.

In the Benshangul-Gumuz region, there has been evidence of a high multidimensional child deprivation index in which 89% of children were found to be deprived in 3 to 6 dimensions as well as a high prevalence of food poverty (24%).^28^ A recent study by UNICEF in the Benshangul-Gumuz region discovered that despite the decline in food poverty from 55% in 1999/2000 to 24% in 2015/2016, only 1.1% of rural households were in the Productive Safety Net Programme compared to 11% of households at the national level.^28^ There is also some evidence of an effect of poor dietary patterns and practices (in younger age groups), and the absence of good water and sanitation practices and facilities in the Benshangul-Gumuz that contribute significantly to childhood stunting.^28^ The 2019 EDHS data also shows that only 2% of households use improved (not shared) sanitation facilities in Benishangul-Gumuz.^24^

Some sociodemographic characteristics were found to be associated with the observed spatial and temporal disparities in stunting. Our findings have linked male children to higher odds of stunting which was previously reported by a study conducted in sub-Saharan Africa that suggests that boys are more likely to be stunted than girls because male children may be more vulnerable to health inequalities than female children in the same age-group.^29^ Older children (ages 24-59 months) were the most at-risk age-group for stunting, as previously reported by various studies.^15,16,30,31^ Thus, timely initiation of supplementary feeding to infants is important to meet their changing nutritional requirements to avert any adverse nutritional status later in life. Also, children with smaller birth sizes were more likely to be stunted before age 5 than average- and larger-sized children similar to findings by several studies in Nepal,^31^ Nigeria,^32^ and Tanzania,^33^ which may be driven by a lack of nutritional supplements during pregnancy and later after birth. Additionally, higher birth order was associated with higher odds of stunting,^34,35^ which may follow from an additional birth placing an economic strain on households,^36^ leading to adverse nutritional status.

Moreover, a short (less than 2 years) birth interval was associated with higher odds of stunting,^37,38^ which may have adverse nutritional implications for the child.^39^ Adequately spaced children may have the necessary nutrition for growth and development and a strong immune system, thereby reducing the likelihood of childhood undernutrition.^40^ Home-born children were more likely to be stunted than those born at a health facility and, thus, access to a health facility may play a crucial role in reducing the stunting disparities. Various studies have linked improved access to skilled delivery care to enhanced maternal and child health^41,42^ likely because mothers may be provided with the relevant information and an understanding of health practices needed to improve their nutritional status and that of their children. As with the present study, children of richer households^16,31,38^ and higher maternal educational attainment^43-47^ have been linked to reduced odds of stunting emphasizing the importance of higher socioeconomic characteristics as a protective factor against adverse child nutritional outcomes. Also, children of underweight mothers were more likely to be stunted than children of normal or overweight mothers, providing support for previous reports of an association between maternal body mass index and odds of under-five stunting.^48-51^

Our findings also highlight sanitation concerns, such as the use of unimproved toilet facilities, which are linked to increased under-five stunting. Water supply and sanitation, given their direct impact on infectious diseases, especially diarrhea, have been considered important for preventing malnutrition^52^ while unsanitary conditions, such as open defecation due to lack of access to improved toilet facilities, have also been linked to childhood stunting.^53^

Furthermore, we found notable residential disparities in under-five stunting as children living in rural settings were more likely to be stunted, which both directly supports^18^ and contrasts^17,29^ findings of previous studies. At the regional contextual level, it appeared that regions with above-average infant mortality rates were associated with higher stunting odds than below-average regions. Therefore, residing in regions with higher infant mortality levels may offer a considerable stunting disadvantage to under-five children. Individual-level research has linked childhood stunting to under-five mortality,^54^ while child undernutrition has in turn been linked to increased child morbidity and mortality risks.^55,56^ Thus, providing a deeper understanding of the under-five and infant mortality nexus may provide crucial future program directions for improving under-five stunting levels in Ethiopia.

This study is not without potential limitations. The cross-sectional nature of the data does not allow for causal inferences. Also, the study did not include some regional aggregate factors which are directly relevant for under-five nutritional status, including food security and nutritional program coverage which were not captured by the survey data. Despite this, the use of a spatial and temporal analytical approach has provided an invaluable contribution to the under-five stunting literature.

## Conclusions

Although childhood stunting has improved in Ethiopia over time, substantial local and regional disparities in under-five stunting remain. Addis Ababa, the capital region, has seen consistently low prevalence, while northern regions have experienced higher levels with slower and inconsistent declines. While Ethiopia may be on track to achieve the WHO’s target of reducing childhood stunting by 40% in 2025, regions especially in the north may not achieve this target.

To ensure continuity across the country, policy decisions should bridge local and regional disparities by focusing on areas that have made slow or uneven progress. Programs and interventions can be targeted to hot spots and high-risk regions found in this study. Moving forward, organizations can project what areas will be at high risk for stunting by identifying regions with larger populations of older under-five children and children from poor households, among other higher risk populations of under-five stunting. Policymakers and stakeholders should also commit resources to improve socioeconomic inequalities among women, as well as household socioeconomic conditions since these underlying factors also impact under-five stunting.

An in-depth investigation into the worsening under-five stunting levels observed in the Dire Dawa region can help to better understand the underlying contextual factors. Future research should consider additional regional-level factors on stunting, including seasonal food insecurity and climatic conditions on stunting. Also, future research can investigate changes in stunting patterns over time using point estimation at smaller spatial scales in Ethiopia.
